# Face-to-face” is not superior to “face-to-screen”: comparing effects of online and offline communication skills course in postgraduate medical students

**DOI:** 10.3389/fmed.2025.1685789

**Published:** 2025-12-11

**Authors:** Wenqi Geng, Jinya Cao, Jiaojiao Hu, Yanping Duan, Tao Li, Yinan Jiang, Lili Shi, Jing Wei

**Affiliations:** Department of Psychological Medicine, Peking Union Medical College Hospital, Chinese Academy of Medical Sciences & Peking Union Medical College, Beijing, China

**Keywords:** empathy, medical students, online education, communication skills, patient-doctor relationship

## Abstract

**Background:**

The integration of online education in medical training has raised questions about its effectiveness for communication skills training (CST), which traditionally relies on face-to-face interaction to cultivate empathy and patient-centered doctor-patient relationship (DPR) orientation. This study aimed to compare the efficacy of online versus offline CST in postgraduate medical students.

**Methods:**

A quasi-experimental pretest-posttest design was conducted with 435 second-year clinical postgraduates, divided into an online group (2022 cohort) and an offline group (2023 cohort). Both groups completed 28 h of CST covering seven communication scenarios, including role-plays and Balint groups. Online sessions used meeting software with mandatory camera use. Empathy was assessed via the Jefferson Scale of Empathy (JSE), and DPR orientation via the Patient-Practitioner Orientation Scale (PPOS). To analyze the effects of the intervention over time and between groups, a two-way repeated-measures ANOVA was conducted.

**Results:**

Both the online and offline groups showed significant increases in PPOS scores after teaching, and the online group had a significant improvement in JSE scores. The main effect of group and the interaction effect between intervention and group on JSE and PPOS were not significant, while the main effect of intervention was significant (JSE: *F* = 6.916, *p* = 0.009; PPOS: *F* = 15.785, *p* < 0.001). Subgroup analyses revealed reduced inter-specialty disparities in patient-centered attitudes post-training, with male and surgical students exhibiting notable shifts toward patient-centeredness.

**Conclusion:**

Online CST yields comparable outcomes to offline training in enhancing empathy and DPR orientation when incorporating interactive elements and visual cues. These findings support integrating online modalities into medical curricula to improve accessibility, with potential for specialty-tailored modules. Further research is needed to explore long-term skill retention.

## 1 Introduction

With the rapid advancement of information technology, medical education has undergone profound transformations, with the concurrent adoption of online and offline teaching models offering diversified pedagogical options (1, 2). Online learning enhances accessibility through anytime-anywhere flexibility, whereas offline instruction preserves the authenticity of face-to-face interaction, enabling students to engage in direct emotional exchanges with instructors and peers (2, 3). The COVID-19 pandemic further accelerated the integration of online teaching into medical curricula, with growing evidence highlighting its potential in knowledge-transmission-focused courses (4, 5). Empirical studies demonstrate that online formats can achieve comparable learning outcomes to traditional offline settings for didactic content, accompanied by high student satisfaction and acceptance rates (1, 6). Encouraging results have also emerged from skills-based training delivered online, though most investigations remain confined to technical skill acquisition (7, 8).

Despite technological advancements allowing virtual face-to-face interactions, online communication inherently risks losing non-verbal cues–such as microexpressions, body language, and paralinguistic signals–compared to in-person encounters (5, 9). This limitation raises questions about the suitability of online models for courses requiring deep interpersonal engagement, particularly communication skills training (CST), where interpersonal interaction and experiential learning are central.

In medical education, CST extends beyond technical proficiencies to include the cultivation of empathy and understanding of the doctor-patient relationship (DPR) (10, 11). Empathy, comprising cognitive (ability to understand others’ perspectives) and emotional (capacity to share others’ feelings) dimensions, is critical for establishing trusting DPRs, improving patient outcomes, and fostering professional resilience (12, 13). The patient-centered DPR model, which emphasizes shared decision-making and alignment with patients’ values, has gained global recognition for its efficacy in enhancing patient satisfaction, treatment adherence, and physician well-being while reducing healthcare costs (14, 15). As the DPR evolves from paternalistic doctor-led models to collaborative partnerships, medical professionals must master the skills to facilitate shared decision-making–a process deeply reliant on empathetic communication.

Communication skills training in postgraduate medical education employs diverse teaching methods, curricular contents, and instructional formats to enhance patient-centered communication (9). Regarding teaching methods, CST integrates didactic and interactive approaches. Didactic methods, including lectures, seminars, and presentations, deliver foundational knowledge. For example, lectures on communication theories or standardized guidelines like the SPIKES model (Set-up, Perception, Invitation, Knowledge, Emotion, and Summary) for breaking bad news are commonly used. Online platforms and videos are increasingly utilized to supplement traditional didactics, offering flexibility for students (16). Interactive methods, however, form the core of skill development. Role-play with feedback sessions is pivotal, allowing students to practice scenarios often with standardized patients or actors (17). Facilitated workshops and group discussions further promote active learning, encouraging peer observation and reflective practice (11). Self-directed role-play and independent group discussions also enable learners to rotate roles (e.g., patient, doctor, observer), fostering introspection and collaborative learning (18). For curricular content, specialty-tailored content is emphasized: oncologists learn prognosis communication, surgeons focus on post-operative discussions, and palliative care trainees master advance care planning (19). Instructional formats vary based on program goals. Short, intensive workshops (1–3 h) target specific skills, ideal for time-constrained trainees. Longitudinal, spiral curricula extend over months or years, progressively introducing complex topics. For instance, a 14-session program for family medicine residents first teaches basic empathy before advancing to bad news delivery (20). Integration into formal residency curricula provides “protected time,” while *ad hoc* sessions (e.g., grand rounds or retreats) offer flexibility. Hybrid models combine in-person workshops with online modules, leveraging technology for accessibility. Assessment methods align with Kirkpatrick’s framework, evaluating reactions, learning, behavior, and patient outcomes through surveys, simulations, and workplace observations. In summary, CST combines didactic clarity with interactive practice, covering both general and specialty-specific skills through flexible formats. This multifaceted approach aims to bridge knowledge gaps and foster adaptive communication essential for effective patient care.

Against this backdrop, the present study evaluates the impact of online versus offline communication skills training on postgraduate medical students’ empathy and DPR orientation. By comparing post-training assessments of these constructs across teaching modalities, we hypothesize that offline “face-to-face” CST confers superior advantages over online “face-to-screen” formats in enhancing postgraduate medical students’ empathy and shaping their doctor-patient relationship orientation, thereby informing evidence-based curriculum design in medical education.

## 2 Materials and methods

### 2.1 Study design and participants

This study employed a quasi-experimental pretest-posttest control group design. A total of 462 medical students were recruited from the Peking Union Medical College between February-April 2022 and February-April 2023. Participants were divided into an experimental group (2022 cohort, online teaching) and a control group (2023 cohort, conventional offline teaching) based on their enrollment year. Eligibility criteria included: second-year postgraduate students in clinical medicine who had clinical internship experience prior to the CST.

Before data collection, all participants received detailed information about the study objectives, procedures, data collection methods, anonymization protocols, and informed consent processes, providing electronic signatures for consent. After consent, participants completed self-administered questionnaires via QR code on mobile devices, with assessments conducted at baseline (pre-intervention) and post-intervention. A total of 435 valid pretest questionnaires and 406 valid posttest questionnaires were obtained. The study protocol was approved by the Ethics Committee of Peking Union Medical College Hospital (I-24ZM0031), with all data reported anonymously following aggregated analysis.

### 2.2 Interventions

#### 2.2.1 Settings

The intervention consisted of a clinical CST delivered over 28 h, structured into seven half-day sessions (4 h each) based on seven doctor-patient communication scenarios/tasks: establishing the patient-doctor relationship, history taking, explaining medical information, negotiating treatment plans, coping with conflicts, breaking bad news, and motivational interviewing. The course lasted for 4 weeks, with each weekly session focusing on a specific communication scenario to ensure progressive skill development and practical application.

Informed by Kolb’s Experiential Learning Theory and peer teaching principles, the course emphasized students’ reflective summarization through practical engagement and capacity building via equal interactive feedback.

Each session included three components, with a 10-min break every hour:

- Theoretical teaching (0.5–1 h): Lectures covered foundational medical psychology, feedback principles, empathy, patient-centered communication concepts, and scenario-specific skills.

- Small-group practice (2–2.5 h): Students engaged in role-playing exercises (patient/doctor roles) using standardized scripts of clinical scenario drama, with peers and instructors acting as observers to provide feedback. Instructors demonstrated and debriefed on challenging communication moments.

- Balint group sessions (1 h): Founded by Michael Balint, the Balint group is a structured peer support forum for healthcare professionals (21). It focuses on exploring emotional dynamics and relational challenges in clinical encounters through case discussions. Participants share real doctor-patient interactions, reflecting on unspoken feelings, power imbalances, and emotional responses without judgment. Facilitated by trained leaders, the group helps members enhance empathy, refine communication skills, and process professional stress.

#### 2.2.2 Group-specific interventions

The control group received the traditional offline teaching model described above. The experimental group received an online teaching model, with all components (lectures, group practices, and Balint sessions) delivered via the Tencent Meeting, an online conferencing software available on both desktop and mobile clients. Both instructors and students were required to keep their cameras on throughout the sessions to ensure facial visibility, while microphones could be either always on or activated during speaking turns.

### 2.3 Measures

#### 2.3.1 Demographic questionnaire

A self-developed demographic questionnaire collected participants’ baseline characteristics, including gender, age, specialty (surgical/non-surgical/technical departments), and years of clinical experience.

#### 2.3.2 Empathy ability

Empathy was measured using the Jefferson Scale of Empathy (JSE) (22), a 20-item self-report questionnaire with a 7-point Likert scale (1 = strongly disagree, 7 = strongly agree), including ten reverse-scored items. Total scores range from 20 to 140, with higher scores indicating greater empathy. Subscale scores were calculated for perspective taking, compassionate care, and walking in patient’s shoes. The scale demonstrated good internal consistency (Cronbach’s α = 0.83) in prior Chinese medical student populations.

#### 2.3.3 Doctor-patient relationship orientation

The Patient-Practitioner Orientation Scale (PPOS) developed by Krupat et al. (23) assesses two dimensions:

- Care: Concern for patients’ personal feelings, life context, and practical challenges.

- Share: Attitudes toward shared decision-making in medical interactions.

The 18-item scale uses a 6-point Likert scale (1 = strongly disagree, 6 = strongly agree), with higher total scores reflecting stronger endorsement of patient-centered communication and lower scores indicating preference for traditional doctor/disease-centered models (23). The scale showed acceptable internal consistency (Cronbach’s α = 0.84) in Chinese medical students sample.

### 2.4 Statistical analysis

Data were analyzed using IBM SPSS 22.0. Quantitative variables were described as mean ± standard deviation while categorical variables were described as frequencies (percentages). The Chi-square test was used to compare the distributions of categorical variables among the groups. Within-group comparisons before and after the intervention were performed using paired *t*-tests, and between-group comparisons at each time point were analyzed using independent *t*-tests. To analyze the effects of the intervention over time and between groups, a two-way repeated-measures ANOVA was conducted. Assumptions for repeated ANOVA were verified using Mauchly’s test of sphericity; when violated, the Greenhouse–Geisser correction was applied. Pearson’s correlation test was used to explore the associations between years of clinical experience and empathy, as well as doctor-patient relationship orientation. A statistical significance level of *p* < 0.05 was applied for all tests.

## 3 Results

### 3.1 Demographic information

The demographic characteristics of the participants are presented in Table 1. The total sample included 435 participants (35.6% male, 64.4% female) with a mean age of 24.98 ± 1.56 years. Participants were distributed across three specialty areas: medical technology (15.4%), non-surgical (39.3%), and surgical (45.3%), with similar proportions observed between the control (offline teaching) and experimental (online teaching) groups. Mean years of clinical experience were 1.65 ± 0.29 years overall. Statistical comparisons revealed no significant differences between groups in age (*p* = 0.998), gender distribution (*p* = 0.127), specialty composition (*p* = 0.879), or clinical experience duration (*p* = 0.894), indicating comparable baseline characteristics across the offline and online teaching groups.

**TABLE 1:** Demographic characteristics of participants.

Characteristics Mean ± SD/*N* (%)	Total	Control/offline	Experimental/online	*P*-value
Age	25.0 ± 1.6	25.0 ± 1.4	25.0 ± 1.6	0.99
**Gender**				
Male	155 (35.6)	83 (32.5)	72 (40.0)	0.13
Female	280 (64.4)	172 (67.5)	108 (60.0)	
**Specialties**				
Medical technology	67 (15.4)	39 (15.3)	28 (15.6)	0.88
Non-surgical	171 (39.3)	98 (38.4)	73 (40.6)	
Surgical	197 (45.3)	118 (46.3)	79 (43.9)	
Years of clinical experience	1.7 ± 0.3	1.7 ± 0.2	1.6 ± 0.3	0.89

### 3.2 Effects of intervention on empathy and doctor-patient relationship orientation among participants

Table 2 presents the pre- and post-intervention results of empathy (JSE) and DPR orientation (PPOS). In the offline (control) group, students’ PPOS scale scores significantly increased compared with pre-intervention; in the online (experimental) group, students showed significant improvements in both JSE and PPOS scale scores. Table 3 presents the pre- and post-intervention comparisons of offline and online groups. Two-way repeated-measures ANOVA reveal that the main effect of group and the interaction effect between intervention and group on JSE and PPOS were not significant, while the main effect of intervention was significant (JSE: *F* = 6.916, *p* = 0.009; PPOS: *F* = 15.785, *p* < 0.001).

**TABLE 2:** Pre- and post-intervention comparisons.

Measures	Control/offline		*t*-value	*P*-value	Experimental/online		*t*-value	*P*-value
	Pre-intervention	Post-intervention			Pre-intervention	Post-intervention		
JSE	107.8 ± 14.8	108.9 ± 16.9	0.89	0.37	106.4 ± 15.5	109.9 ± 16.7	2.85	**0.01**
JSE-W	34.3 ± 4.8	34.9 ± 5.4	1.30	0.20	34.0 ± 5.0	35.1 ± 4.9	2.53	**0.01**
JSE-C	17.5 ± 4.7	17.2 ± 5.0	0.55	0.58	17.5 ± 4.9	17.8 ± 5.4	0.73	0.47
JSE-P	56.0 ± 7.9	56.8 ± 8.3	1.33	0.19	54.9 ± 8.3	56.9 ± 8.1	3.25	**0.001**
PPOS	59.5 ± 8.8	63.2 ± 12.9	3.66	**<0.001**	60.9 ± 12.1	63.1 ± 14.0	2.04	**0.04**
PPOS-C	27.3 ± 4.9	29.1 ± 7.3	3.17	**0.002**	27.9 ± 6.8	29.5 ± 7.8	2.59	**0.01**
PPOS-S	32.2 ± 4.8	34.0 ± 6.3	3.64	**<0.001**	33.0 ± 6.3	33.6 ± 7.0	1.11	0.27

Bold values indicate *p* < 0.05. JSE, Jefferson Scale of Empathy; JSE-W, walking in patient’s shoes subscale of JSE; JSE-C, compassionate care subscale of JSE; JSE-P, perspective taking subscale of JSE; PPOS, Patient-Practitioner Orientation Scale; PPOS-C, caring subscale of PPOS; PPOS-S, sharing subscale of PPOS.

**TABLE 3:** Comparisons between offline and online teaching.

Group	Measures	Pre-intervention	Post-intervention	Mean square	*F*-value	*P*-value	Partial η^2^
Online	JSE	106.4 ± 15.5	109.9 ± 16.7				
Offline		107.8 ± 14.8	108.9 ± 16.9				
			Group main effect	8.5	0.02	0.88	<0.001
			Intervention main effect	803.4	6.92	**0.01**	0.02
			Group × intervention	210.8	1.82	0.18	0.01
Online	PPOS	60.9 ± 12.1	63.1 ± 14.0				
Offline		59.5 ± 8.8	63.2 ± 12.9				
			Group main effect	68.4	0.32	0.57	0.001
			Intervention main effect	1328.0	15.80	**<0.001**	0.05
			Group × intervention	83.7	1.00	0.32	0.003

Bold values indicate *p* < 0.05. JSE, Jefferson Scale of Empathy; PPOS, Patient-Practitioner Orientation Scale.

### 3.3 Factors influencing empathy and doctor-patient relationship orientation among participants

There were no significant correlations between years of clinical experience and scores on either the JSE or the PPOS. Changes in empathy and DPR orientation scores by gender and specialty before and after the training were presented in the Supplementary Tables 1, 2. Notably, male students had higher PPOS scores, with significant between-gender differences observed in the offline group (Pre-intervention: Male 62.70 ± 9.45 vs. Female 60.28 ± 8.54, *t* = 2.04, *p* = 0.042, Post-intervention: Male 65.52 ± 16.1 vs. Female 60.15 ± 11.20, *t* = 2.47, *p* = 0.014). In the online group, pre-intervention PPOS scores varied significantly by specialty (*F* = 3.063, *p* = 0.049), with a notable difference between non-surgical and surgical specialties (Non-surgical: 63.37 ± 14.07 vs. Surgical: 58.71 ± 10.59, *p* = 0.014). This inter-specialty difference became non-significant post-intervention. No significant specialty-related differences were observed in the offline group for DPR orientation scores.

## 4 Discussion

The present study compared the effects of online and offline CST on empathy and doctor-patient relationship (DPR) orientation among postgraduate medical students. After both online and offline CST, students’ DPR orientation shifted toward patient-centeredness, while online CST was able to enhance students’ empathy levels. Contrary to the hypothesis that face-to-face offline interaction would yield superior outcomes, the outcomes of offline and online teaching were comparable. After offline communication training, male medical students showed a greater increase in patient-centered tendency. We further observed that initially evident inter-specialty discrepancies in patient-centered DPR attitudes were mitigated following training. These finding underscore the potential of structured communication curricula to address discipline-specific biases and gender differences in patient-centered care orientation among medical students.

Before the pandemic, educators had already explored the effectiveness of online teaching for medical students’ communication skills, empathy, and shared decision-making abilities. Kyaw et al. conducted a systematic review and meta-analysis in 2019 of ten RCTs and two cluster RCTs, concluding that–despite low-quality evidence–digital education was as effective as traditional learning in training medical students’ communication skills (9). They also found no post-intervention differences between more or less interactive forms of digital education. Post-pandemic, the popularity of online teaching has increased, leading to more educational studies adopting online teaching formats, including those focused on CST. We have summarized some teaching studies related to CST and our present study, which are presented in Supplementary Table 3 (5, 15, 16, 24–27). In summary, when teaching methods include interactive components–whether VR-based interaction [Kron et al. (24)], video-based interaction [Kaltman et al. (25)], or offline interpersonal interaction [Lanken et al. (16)]–the teaching can effectively enhance communication competencies. In contrast, for pure lectures [Shen et al. (5)] or non-interactive multimedia-based teaching [Chittenden et al. (26)], there is no significant difference in the improvement of communication skills between online and offline formats, or offline teaching outperforms online teaching. In our study, both teaching models integrated role-playing activities and Balint groups, which might have alleviated the deficit of in-person interaction in the online setting. The mandatory use of cameras in the online group could have preserved visual cues (e.g., facial expressions), which are essential for cultivating empathy. Comparing our results with the above findings, it can be inferred that when the teaching content and design are identical and include interactive communication components, online and offline teaching formats may yield comparable effects in enhancing medical students’ communication skills.

In the subgroup analysis, with offline teaching, male medical students showed a greater increase in patient-centered tendency, while with online teaching, students in surgical departments showed significant changes in DPR orientation scores, indicating that the teaching content was not equally effective for all students. We have not identified any previous studies with similar conclusions. Although the content of online and offline teaching was the same and their formats strived to be consistent, we speculate that there may be differences in individual learning outcomes among students, which might be influenced by gender and specialty factors. Future research with larger sample sizes and diverse teaching settings may be needed to clarify whether this trend has generalizability. This highlights the need for refined and differentiated communication teaching tailored to different students.

Our study provides robust data for the development of communication teaching for medical students and fills a gap in the scarce Asian medical education research in this field. However, the study has several limitations. First, non-random assignment (by enrollment year) introduces selection bias. Although baseline demographics were balanced, unmeasured variables (e.g., learning motivation) may have influenced the results. Second, we used two self-rated questionnaires, the JSE and PPOS, to assess medical students’ empathy and doctor-patient relationship orientation, which could be potentially skewed by social desirability bias. Additionally, we did not conduct post-course follow-ups with medical students, making it impossible to determine whether the similar communication skills in the online and offline teaching groups could be sustained.

This study’s findings support integrating online CST into medical curricula, challenging the primacy of offline formats. Policymakers and educators can leverage online modalities to enhance accessibility for time-constrained or geographically limited trainees, with mandatory camera use preserving critical visual cues for empathy development. The mitigation of disparities in patient-centered attitudes post-training highlights the need for specialty- or individual-tailored CST. Hybrid models combining online flexibility with offline interactivity may optimize outcomes, while targeted online modules could effectively strengthen specific patient care dimensions.

A critical unanswered question is whether the comparable effects of online and offline communication training can be sustained long-term. Longitudinal follow-ups (6–12 months) are essential to determine if gains persist in clinical practice. Additionally, the mechanisms behind online-offline equivalence need exploration–qualitative work (e.g., focus groups) could clarify which factors compensate for reduced non-verbal cues. Learner traits like digital literacy may moderate outcomes, requiring investigation into targeted support. Larger randomized multi-center studies across training levels and systems are needed to validate findings and generalize recommendations.

## 5 Conclusion

Our study challenges the assumption that offline education is inherently superior in cultivating empathy and patient-centered communication. Our findings advocate for the evidence-based integration of online methods into medical education, with targeted adjustments tailored to specialty-specific needs. Future research should focus on optimizing the design of communication curricula for medical students, as well as conducting follow-up observations on their empathy and doctor-patient relationship orientation.

## Data Availability

The raw data supporting the conclusions of this article will be made available by the authors, without undue reservation.
