# Supplementary material for: Face-to-face” is not superior to “face-to-screen”: comparing effects of online and offline communication skills course in postgraduate medical students
Source: Front Med (Lausanne). 2025 Dec 11;12:1685789. doi: 10.3389/fmed.2025.1685789 (PMC12738318; doi:10.3389/fmed.2025.1685789)
Supplement: Supplementary file 1 [file Data_Sheet_1.docx]

Supplementary Material

# Supplementary Data

Table S1. Empathy and doctor-patient relationship orientation in different gender

Table S2. Empathy and doctor-patient relationship orientation in different specialties

Table S3. Summary of recent research on online CST

Table S1. Empathy and doctor-patient relationship orientation in different gender

|  | **Measures** | **Control / Offline** | | **t value** | **p value** | **Experimental / Online** | | **t value** | **p value** |
| --- | --- | --- | --- | --- | --- | --- | --- | --- | --- |
|  |  | **Male (83)** | **Female (172)** |  |  | **Male (72)** | **Female (108)** |  |  |
| **Pre-intervention** | JSE | 106.7 ± 15.2 | 108.8 ± 14.2 | -1.14 | 0.25 | 106.9 ± 15.6 | 109.2 ± 14.1 | -0.99 | 0.32 |
|  | JSE-W | 55.4 ± 8.2 | 56.5 ± 8.0 | -1.01 | 0.31 | 55.2 ± 8.3 | 56.0 ± 8.0 | -0.67 | 0.50 |
|  | JSE-C | 34.3 ± 5.2 | 34.5 ± 4.8 | -0.27 | 0.79 | 34.8 ± 4.6 | 34.5 ± 4.6 | 0.38 | 0.70 |
|  | JSE-P | 16.9 ± 5.1 | 17.8 ± 4.2 | -1.60 | 0.11 | 17.0 ± 5.7 | 18.7 ± 4.1 | -2.29 | **0.02** |
|  | PPOS | 62.70± 9.5 | 60.3 ± 8.5 | 2.05 | **0.04** | 62.8 ± 14.5 | 59.6 ± 9.4 | 1.77 | 0.08 |
|  | PPOS-C | 29.0 ± 5.7 | 27.7 ± 4.7 | 1.92 | 0.06 | 29.4 ± 8.1 | 26.8 ± 5.3 | 2.64 | **0.01** |
|  | PPOS-S | 33.8 ± 5.0 | 32.6 ± 4.8 | 1.74 | 0.08 | 33.4 ± 7.2 | 32.8 ± 5.2 | 0.57 | 0.57 |
| **Post-intervention** | JSE | 104.3 ± 19.1 | 109.0 ± 15.6 | -1.69 | 0.09 | 106.2 ± 18.1 | 107.7 ± 16.5 | -0.68 | 0.50 |
|  | JSE-W | 54.5 ± 9.0 | 56.8 ± 7.8 | -1.70 | 0.09 | 55.2 ± 8.9 | 55.8 ± 8.0 | -0.56 | 0.58 |
|  | JSE-C | 33.8 ± 5.7 | 34.9 ± 5.2 | -1.26 | 0.21 | 34.1 ± 5.3 | 34.3 ± 5.1 | -0.17 | 0.86 |
|  | JSE-P | 16.0 ± 6.1 | 17.2 ± 4.6 | -1.53 | 0.13 | 16.8 ± 5.8 | 17.6 ± 5.2 | -1.12 | 0.26 |
|  | PPOS | 65.5 ± 16.1 | 60.2 ± 11.3 | 2.50 | **0.01** | 63.5 ± 15.0 | 62.1 ± 14.2 | 0.70 | 0.48 |
|  | PPOS-C | 30.6 ± 9.2 | 27.9 ± 6.2 | 2.24 | **0.03** | 30.1 ± 8.2 | 28.8 ± 8.0 | 1.17 | 0.24 |
|  | PPOS-S | 34.9 ± 7.3 | 32.3 ± 5.8 | 2.54 | **0.01** | 33.4 ± 7.6 | 33.3 ± 6.8 | 0.11 | 0.92 |

Abbreviations: JSE, Jefferson Scale of Empathy. JSE-W, Walking in patient's shoes subscale of JSE. JSE-C, Compassionate care subscale of JSE. JSE-P, Perspective taking subscale of JSE. PPOS, Patient-Practitioner Orientation Scale. PPOS-C, Caring subscale of PPOS. PPOS-S, Sharing subscale of PPOS.

Table S2. Empathy and doctor-patient relationship orientation in different specialties

|  | **Measures** | **Control / Offline** | | | **F value** | **p value** | **Experimental / Online** | | | **F value** | **p value** |
| --- | --- | --- | --- | --- | --- | --- | --- | --- | --- | --- | --- |
|  |  | **Surgical (118)** | **Nonsurgical (98)** | **Technical (39)** |  |  | **Surgical (79)** | **Nonsurgical (73)** | **Technical (28)** |  |  |
| **Pre-intervention** | JSE | 107.9 ± 14.2 | 108.2 ± 14.8 | 108.3 ± 15.2 | 0.02 | 0.98 | 108.6 ± 14.1 | 107.5 ± 16.0 | 109.5 ± 13.4 | 0.22 | 0.80 |
|  | JSE-W | 55.8 ± 8.0 | 56.2 ± 8.1 | 57.1 ± 8.2 | 0.35 | 0.71 | 56.1 ± 7.8 | 55.2 ± 8.7 | 55.9 ± 7.5 | 0.26 | 0.77 |
|  | JSE-C | 34.3 ± 4.9 | 34.6 ± 4.9 | 34.3 ± 5.1 | 0.14 | 0.87 | 34.6 ± 4.8 | 34.6 ± 4.6 | 34.7 ± 3.9 | 0.01 | 0.99 |
|  | JSE-P | 17.8 ± 4.3 | 17.4 ± 4.7 | 17.0 ± 4.8 | 0.55 | 0.58 | 14.9 ± 4.5 | 17.7 ± 5.5 | 18.9 ± 3.6 | 0.57 | 0.56 |
|  | PPOS | 61.4 ± 8.8 | 60.5 ± 8.4 | 61.7 ± 10.4 | 0.39 | 0.68 | 58.7 ± 10.6 | 63.4 ± 14.1 | 60.6 ± 5.7 | 3.06 | **0.049** |
|  | PPOS-C | 28.2 ± 5.1 | 27.8 ± 4.9 | 28.5 ± 5.4 | 0.39 | 0.68 | 26.9 ± 5.9 | 29.1 ± 8.0 | 27.6 ± 3.98 | 2.12 | 0.12 |
|  | PPOS-S | 33.2 ± 4.9 | 32.7 ± 4.4 | 33.1 ± 5.8 | 0.27 | 0.76 | 31.9 ± 5.9 | 34.3 ± 6.9 | 33.0 ± 3.2 | 3.19 | **0.04** |
| **Post-intervention** | JSE | 106.3 ± 16.7 | 109.3 ± 16.6 | 105.8 ± 18.9 | 0.66 | 0.52 | 106.7 ± 18.6 | 106.1 ± 15.3 | 110.0 ± 17.5 | 0.79 | 0.45 |
|  | JSE-W | 55.7 ± 8.3 | 56.7 ± 8.0 | 55.3 ± 9.1 | 0.44 | 0.65 | 55.7 ± 9.0 | 54.8 ± 7.4 | 56.7 ± 8.4 | 0.75 | 0.47 |
|  | JSE-C | 34.2 ± 5.2 | 34.9 ± 5.4 | 34.8 ± 6.1 | 0.30 | 0.74 | 34.1 ± 5.3 | 33.9 ± 4.7 | 35.1 ± 5.5 | 0.76 | 0.47 |
|  | JSE-P | 16.5 ± 5.0 | 17.7 ± 5.2 | 15.7 ± 5.5 | 1.62 | 0.20 | 16.9 ± 5.7 | 17.3 ± 5.1 | 18.2 ± 5.4 | 0.94 | 0.39 |
|  | PPOS | 62.5 ±13.8 | 60.5 ±11.9 | 62.0 ± 13.3 | 0.74 | 0.48 | 62.4 ± 15.6 | 63.7 ± 13.4 | 61.2 ± 13.8 | 0.47 | 0.62 |
|  | PPOS-C | 29.1 ± 7.6 | 28.0 ± 6.6 | 30.2 ± 9.1 | 0.91 | 0.41 | 29.5 ± 8.6 | 29.4 ± 7.5 | 28.7 ± 7.7 | 0.17 | 0.84 |
|  | PPOS-S | 33.4 ± 6.8 | 32.6 ± 5.9 | 33.8 ± 6.5 | 0.46 | 0.63 | 32.9 ± 7.6 | 34.3 ± 6.6 | 32.5 ± 6.6 | 1.28 | 0.28 |

Abbreviations: JSE, Jefferson Scale of Empathy. JSE-W, Walking in patient's shoes subscale of JSE. JSE-C, Compassionate care subscale of JSE. JSE-P, Perspective taking subscale of JSE. PPOS, Patient-Practitioner Orientation Scale. PPOS-C, Caring subscale of PPOS. PPOS-S, Sharing subscale of PPOS.

Table S3. Summary of recent research on online CST

| **Authors** | **Subjects** | **Teaching modalities and settings** | **Primary outcomes / Measures** | **Results** |
| --- | --- | --- | --- | --- |
| Lanken et al. (2015)[16] | 129 residents | Control group: usual curricula  Intervention group: usual curricula + 1-hour self-directed viewing module + 1-hour small-group debriefing | Communication skills (JSPE) | Intervention group higher scores |
| Gartmeier et al. (2015)[27] | 72 medical students and 96 student teachers | Equal learning time of 300 min:  (a) e-learning with video cases and roleplay with video feedback combined  (b) only e-learning with video cases  (c) only role-play with video feedback  (d) a wait-list control group | Communication skills (relationship, problem-solving, structures, etc.) | Combined condition was more effective than e-learning and  role-play alone |
| Kron et al. (2017)[24] | 421 2^nd^-year medical students | VR: Multimedia presentation, virtual interaction  Computer based: Illustrative videos | Communication skills (openness/defensiveness, collaborative/competitive, nonverbal communication, presence) | VR group: significantly higher composite scores |
| Chittenden et al. (2017) [26] | 121 3^rd^-year medical students | 3-hour workshop  Classic web-based: video  Multimedia web-based: video (music and text, quicker pace)  Written: paper content | Communication skills (checklist) | No differences between groups |
| Kaltman et al. (2018) [25] | 99 1^st^-year medical students | Standard: class instruction  Simulation: class instruction + 3 weeks of 3 interactive video simulations | Communication skills (open- and closed-ended questions, reflections, empathic responses) | Simulation group: more open-ended questions and empathic responses |
| Lin et al. (2024) [15] | 323 4^th^-year medical students | 3 hours, theory + role play  Offline: web platform  Online: classroom | Decision making (COMRADE), communication skills(MPI) | Both groups: Significant improvement in SDM proficiency and communication skills |
| Shen et al. (2025) [5] | 171 4^th^-year neurosurgery students | 5 lectures  Online: web platform  Offline: classroom | Communication skills (self report, 5-Likert scale) | Offline group: better communication skills |
| Our present study (2025) | 462 2^nd^-year medical students | 28 hours, theory + role-play + Balint groups  Online: web platform  Offline: classroom | Communication skills (JSE, PPOS) | Both groups: improvement in PPOS  Online: improvement in JSE |

Abbreviations: JSE: Jefferson Scale of Empathy. VR: Virtual Reality. COMRADE: Combined Outcome Measure for Risk Communication and Treatment Decision Making Effectiveness. MPI: Matched‑Pair Instrument in Doctor‑Patient Communication Skills. SDM: Shared Decision Making. PPOS: Patient-Practitioner Orientation Scale.
